# Zein as an Effective Carrier for Hesperidin Delivery Systems with Improved Prebiotic Potential

**DOI:** 10.3390/molecules28135209

**Published:** 2023-07-04

**Authors:** Szymon Sip, Anna Sip, Andrzej Miklaszewski, Marcin Żarowski, Judyta Cielecka-Piontek

**Affiliations:** 1 Department of Pharmacognosy and Biomaterials, Poznan University of Medical Sciences, Rokietnicka 3, 60-806 Poznan, Poland; szymonsip@ump.edu.pl; 2 Department of Biotechnology and Food Microbiology, Poznan University of Life Sciences, Wojska Polskiego 48, 60-627 Poznan, Poland; anna.sip@up.poznan.pl; 3 Institute of Materials Science and Engineering, Poznan University of Technology, Jana Pawła II 24, 61-138 Poznan, Poland; andrzej.miklaszewski@put.poznan.pl; 4 Department of Developmental Neurology, Poznan University of Medical Sciences, Przybyszewski 49, 60-355 Poznan, Poland; zarowski@ump.edu.pl

**Keywords:** hesperidin, zein, extended-release, improved solubility, microbiome, *Lactiplantibacillus*

## Abstract

Hesperidin is a polyphenol derived from citrus fruits that has a broad potential for biological activity and the ability to positively modify the intestinal microbiome. However, its activity is limited by its low solubility and, thus, its bioavailability—this research aimed to develop a zein-based hesperidin system with increased solubility and a sustained release profile. The study used triple systems enriched with solubilizers to maximize solubility. The best system was the triple system hesperidin-zein-Hpβ-CD, for which the solubility improved by more than six times. A significant improvement in the antioxidant activity and the ability to inhibit α-glucosidase was also demonstrated, due to an improved solubility. A release profile analysis was performed in the subsequent part of the experiments, confirming the sustained release profile of hesperidin, while improving the solubility. Moreover, the ability of selected probiotic bacteria to metabolize hesperidin and the effect of this flavonoid compound on their growth were investigated.

## 1. Introduction

The mutual relationship between natural substances of plant origin and the gut microbiota is becoming increasingly attractive in the scientific community [1,2,3]. A significant part of active compounds derived from plants are characterized by low bioavailability, due to their low solubility and low permeability through cell membranes, due to a high molecular weight [4]. Often, activity is observed after the intestinal microbiota have activated a given compound, often modifying the structure by cutting off the sugar residue, creating an aglycon with a significantly lower molecular weight and, thus, a significantly higher ability to penetrate the cell membrane [5,6]. On the other hand, active compounds affect the intestinal microbiota composition [7,8]. It seems that the action of active compounds in the human organism depends on the state of the intestinal microbiome.

On the one hand, mutual interactions are crucial, in order to maximize the therapeutic potential of natural active compounds [7,9]. In addition, due to the intestinal microbiome’s complexity and its multiplicity of functions, the possibility of beneficial modification of its profile by administering natural active compounds may prove crucial in developing new non-invasive therapies [10,11,12,13].

Hesperidin (HES) belongs to the group of flavonoid compounds. Its primary sources in natural food are citrus fruits, mainly in the white subcutaneous layer, albedo. HES, as a glycoside, is a large molecule of low solubility with poor permeability through cell membranes, which translates into a low bioavailability [14,15,16]. Unfortunately, a low bioavailability determines limited biological activity. One of the methods to improve the bioavailability of sparingly soluble active substances is to improve their solubility through methods widely described in the literature, primarily based on the preparation of polymer systems, which result in an increase in the wetting surface of the dissolved molecule, conditioning its better solubility [17,18,19]. In the case of HES, an improvement in solubility alone will not translate into a significant increase in bioavailability, due to a low permeability through cell membranes [20,21]. HES can be metabolized in the gastrointestinal tract to the hesperetin (HEP) molecule, an aglycone. Such a conversion can only occur under the gut microbiota, producing the enzyme hesperidin 6-*O*-α-l-rhamnosyl-β-d-glucosidase capable of hydrolyzing the bond [22,23,24].

HES is characterized by a broad biological activity, ranging from the antioxidant activity characteristic of polyphenols, through antidiabetic activity, to antitumor and neuroprotective activity [25,26,27,28]. However, its potential use in treating diabetes seems particularly interesting, due to the connection of HES metabolism with the intestinal microbiome. The intestinal microbiome plays an essential role in the course and development of type 2 diabetes [29,30]. Thus, using a molecule capable of positively influencing the intestinal microbiome, with a simultaneous metabolism dependent on the qualitative and quantitative composition of the microflora, is a fascinating possibility.

In vivo studies have confirmed the positive effect of HES on the condition of the intestinal microbiome [24,31,32]. Most research used orange juice as the source and carrier of HES. The conducted research focused on the final effect of the impact of HES on quantitative and qualitative changes in the gut microbiome. However, due to the complexity of the microbiome itself, the direct mechanism of action has not been explained. The conducted experiments demonstrated the ability of HES to increase the number of probiotic bacteria, with a simultaneous reduction in the number of potentially pathogenic microorganisms [33,34,35,36]. The series of experiments proposed by the authors aimed to improve the properties of HES itself and explain the mechanisms behind prebiotic abilities.

Zein was used in this work as the base carrier, which is a protein obtained from maize. It is one of the best-known proline-rich plant proteins [37]. It is characterized by being safe to use, not only in the pharmaceutical industry, but also in the food industry, so we were confident about its use as a carrier for orally administered systems [38]. It is characterized by a lack of solubility in water, which determines its use in developing new films for external use and biodegradable polymers intended, for example, for packaging food. Notably, it is characterized by a controlled release of active substances, which, when used orally, allows for the development of a formulation with a significantly prolonged release profile of active substances [39]. In addition, it has antibacterial properties [40]. In our study, zein was chosen as the carrier material for the delivery of hesperidin, due to its unique properties and potential benefits. The selection of zein was based on two primary premises.

First, zein offered the possibility of achieving sustained-release systems, as discussed earlier. This sustained-release property is advantageous in the context of drug delivery systems, as it allows for a controlled and prolonged release of the active compound [41]. In our study, the sustained release of hesperidin from a zein-based system was expected to have a positive impact on the gut microbiota. Previous research has shown that the modulation of release kinetics can influence the composition and activity of the gut microbiome. By utilizing zein as a carrier, we aimed to exploit its sustained-release capability to potentially enhance the prebiotic potential and achieve a favorable impact on the gut microbiota. The discussion segment presented in the revised manuscript will provide further support for this assumption.

Second, the selection of zein was driven by its potential as a source of nutrients for probiotic bacteria, thus providing an additional prebiotic effect. Zein, being a plant protein, has been reported to possess nutritional properties that can support the growth and activity of probiotic strains. This characteristic of zein aligns with our aim to develop a delivery system that not only delivers the active compound but also provides a nourishing environment for probiotic bacteria [42]. By incorporating zein into the formulation, we intended to create a symbiotic relationship between the carrier material and the probiotics, potentially enhancing the overall efficacy and impact of the system.

This work focuses on preparing delivery systems for HES, based on zein as a carrier. Moreover, the interactions between HES and the gut microbiome, to estimate mutual relationships, were determined.

## 2. Results and Discussion

**Figure 1:**
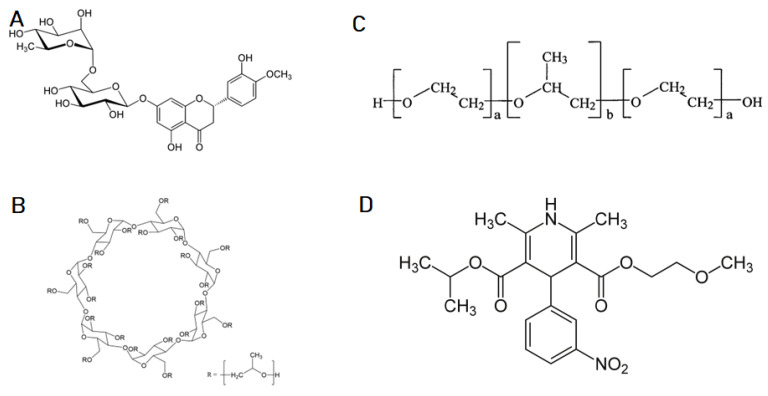
Structural formulas: (**A**) Hesperidin, (**B**) 2-Hydroxypropyl-β-cyclodextrin, (**C**) Pluronic F127, (**D**) Soluplus.

The obtained triple systems containing zein, hesperidin, and one of the selected solubilizers (Soluplus, Pluronic F-127, and Hpβ-CD) (Figure 1) were subjected to PXRD analysis, to investigate the potential for crystallographic changes (Supplementary Materials, Figure S1).

The obtained diffractograms confirmed the amorphous structure of the solubilizers used, while presenting the crystal structure of HES. Characteristic peaks for HES were observed at diffraction angles (2θ) of 12.08°, 15.51°, 19.65°, 21.23°, and 22.35° [43,44]. The analysis confirmed the preservation of the crystalline structure of HES after preparing its binary and triple systems. However, we observe a decrease in the intensity of the characteristic peaks, which proved the dispersion of the crystalline HES in the amorphous solubilizer matrix. Despite not obtaining complete amorphous dispersion from the diffractograms, we concluded that the best dispersion of the active substance was in the Hpβ-CD matrix (Figure S1D) [45,46]. The most substantial reduction in the intensity of the hesperidin peaks was observed; with a possible explanation for this being the penetration of a part of the active substance into the inner chamber of the cyclodextrin and binding, conditioning a better dispersion of HES in the matrix, forming inclusion complexes [47,48].

A Fourier transform infrared spectroscopy (FT-IR) analysis was the next step in determining the physical changes in the obtained systems. The spectra obtained are presented in Figure S2. The FT-IR spectrum of hesperidin usually exhibits a broad and intense peak at 3200–3600 cm^−1^, corresponding to the O–H stretching vibration of the hydroxyl groups present in the flavonoid ring. This peak is often accompanied by a smaller peak at around 1650 cm^−1^, which is associated with the C=C stretching vibration of the aromatic ring. Other notable peaks in the hesperidin FT-IR spectrum include those at around 1600–1700 cm^−1^, which correspond to the carbonyl group of the flavonoid ring, and those at around 1300–1400 cm^−1^, which are attributed to C–O stretching vibrations. There may also be peaks at around 2900–3000 cm^−1^, corresponding to C–H stretching vibrations. The FT-IR spectrum of HP-β-CD usually exhibits a broad peak in the range 3300–3600 cm^−1^, corresponding to the hydroxyl groups’ O–H stretching vibration in the glucose units.

Additionally, a peak at around 2900–3000 cm^−1^ corresponds to C–H stretching vibrations. Other notable peaks in the HP-β-CD FT-IR spectrum include those at around 1630–1650 cm^−1^, which correspond to the C=O stretching vibration of the carbonyl group in the glucose units. There may also be peaks at around 1150–1180 cm^−1^, which are associated with the C–O–C stretching vibrations of the glycosidic bonds linking the glucose units. The FT-IR spectrum of Soluplus shows several characteristic peaks. A broad and strong peak at around 3400 cm^−1^ corresponds to the stretching vibration of hydroxyl groups (-OH) and indicates the presence of hydrogen bonding in the molecule. The peak at around 2925 cm^−1^ is attributed to the stretching vibration of –CH_2_– groups. The peak at around 1740 cm^−1^ corresponds to the stretching vibration of the carbonyl group (C=O). The band at around 1455 cm^−1^ is assigned to the symmetric bending of –CH_2_– groups. The peak at around 1260 cm^−1^ corresponds to the stretching vibration of the ether group (–C–O–C–), and the peak at around 1100 cm^−1^ is attributed to the stretching vibration of the C–O group. Pluronic F127 is a triblock copolymer composed of poly(ethylene oxide)-poly(propylene oxide)-poly(ethylene oxide) (PEO-PPO-PEO) units. Its FT-IR spectrum shows characteristic peaks at around 2880–2840 cm^−1^ (C–H stretching vibrations of PPO), 1465 cm^−1^ (C–H bending vibrations of PPO), 1100–1150 cm^−1^ (C–O–C stretching vibrations of PEO), and 840–920 cm^−1^ (C–H rocking vibrations of PPO). The peak at 2900 cm^−1^ corresponds to the C–H stretching vibrations of PEO. The peak at 1260–1280 cm^−1^ corresponds to PEO’s ether bond (C–O–C) stretching vibration. The broad peak at around 3440 cm^−1^ corresponds to the O–H stretching vibration of the terminal hydroxyl group of PEO. The FT-IR spectrum of zein shows several characteristic peaks, indicating the presence of different functional groups. The broad band observed in the range of 3400–3200 cm^−1^ corresponds to the stretching vibration of the N–H and O–H groups. The peak at 1650 cm^−1^ indicates the presence of the C=O stretching vibration of amide groups, while the peak at 1540 cm^−1^ represents the N–H bending vibration of amide groups. The peak at 1235 cm^−1^ corresponds to the C–N stretching vibration of amide groups, and the peak at 1020 cm^−1^ corresponds to the C–H bending vibration of the alkyl groups. The FT-IR spectrum of zein also shows peaks at 2920–2850 cm^−1^, indicating the presence of methylene groups, which are also observed in other proteins.

The FT-IR spectrum analysis of the double and triple systems of hesperidin with solubilizers did not reveal the presence of any new bands or shifts of bands characteristic of the individual compounds. This indicates that there were no new chemical interactions between hesperidin and the applied solubilizers. However, a decrease in the intensity of the bands characteristic of hesperidin was observed in the double and triple systems, which can be attributed to the dispersion of the solubilizers, resulting in an improved solubility of hesperidin. This indicates that the solubilizers effectively dispersed the hesperidin molecules, resulting in a higher solubility of the compound in the system. The lack of new bonds between HES and HpβCD in the FT-IR spectrum suggests that the formation of an inclusion complex between these two compounds was minimal.

However, it was still possible that the complex was present, but the amount was too small to be detected through FT-IR analysis. Overall, the XPRD and FT-IR characterization results indicated that the HES and solubilizers were physically mixed and dispersed in a matrix, rather than chemically bonded or complexed [49].

The biggest problem with the therapeutic effect of naturally derived active substances is their low solubility in water [50,51]. In the case of HES, we observed a negligible solubility in water, translating into a low therapeutic effect [52]. Hence, a common path in developing new delivery systems is to improve the solubility of sparingly soluble active biological compounds. For this reason, the first stage of the research, aimed at characterizing the properties, was to determine the change in HES solubility in the obtained systems. Zein was selected as the carrier, characterized as improving the solubility of sparingly soluble compounds and providing a sustained release profile [53,54]. Due to its properties, it was possible to obtain a system with an improved dissolution rate, while ensuring a slow release of HES; the obtained systems provided a more prolonged biological activity. A significant positive effect was obtained for all the solubilizers used in the double and triple systems (Table 1). In the case of the solubilizers used, the binary system was better for the system containing Pluronic F127 than the other combinations. In the other obtained systems, zein improved the solubility, which translated into a correspondingly higher concentration of HES in triple systems. The most significant improvement in solubility was obtained for the triple system containing HES-zein-Hpβ-CD (0.3130 mg/mL), representing an over six-fold increase in the solubility of HES. Cyclodextrins are substances with an extraordinary ability to improve the solubility of sparingly soluble compounds, while ensuring safe use [55,56,57]. In addition, their positive prebiotic effect on the gut microbiome has been shown, which was crucial for how subsequent research was conducted [58,59].

**Figure 2:**
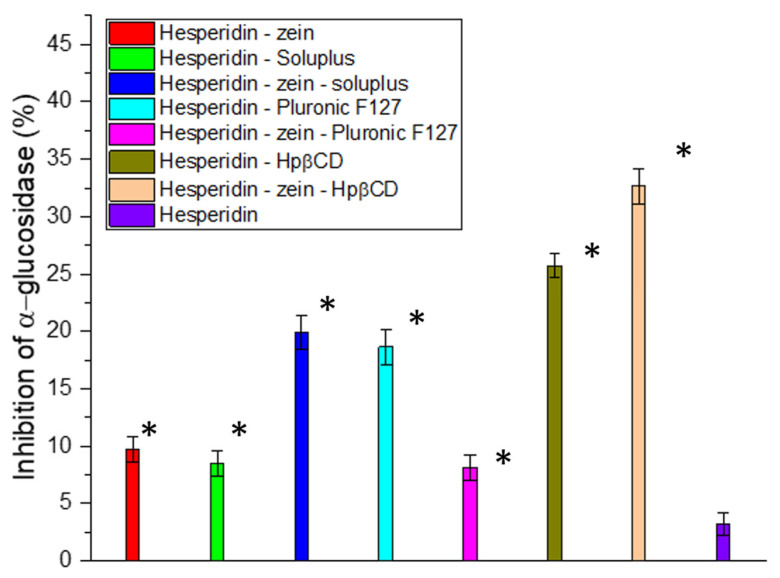
Inhibition of α-Glucosidase by hesperidin, and the resulting double and triple systems. Data expressed as mean ± SD; *—significance with *p* ≤ 0.05.

The next stage of the work was to investigate the influence of the improvement in solubility on the biological activity. Determining the characteristics of the changes was essential, due to the not always linear translation of compound concentration to activity, particularly in the study of enzymatic inhibition activity [60,61]. Due to studies showing a positive effect of using HES in type 2 diabetes, the ability to inhibit α-glucosidase, which determines the breakdown of carbohydrates into simple sugars, was investigated [62,63,64]. The results indicated a high correlation between the obtained concentrations and the degree of enzyme inhibition. Our studies obtained a deficient inhibitory activity at only 3% for pure HES. In the case of the system with the highest solubility, the obtained concentration determined α-glucosidase inhibition at over 32% (Figure 2). Thus, thanks to the 6-fold improvement in solubility, a more than 10-fold increase in the activity associated with enzymatic inhibition was achieved. In the study, no results were obtained that would allow for the determination of IC_50_ for α-glucosidase; however, to estimate the target value, an experiment was performed with correspondingly higher concentrations of HES (Figure S3).

**Figure 3:**
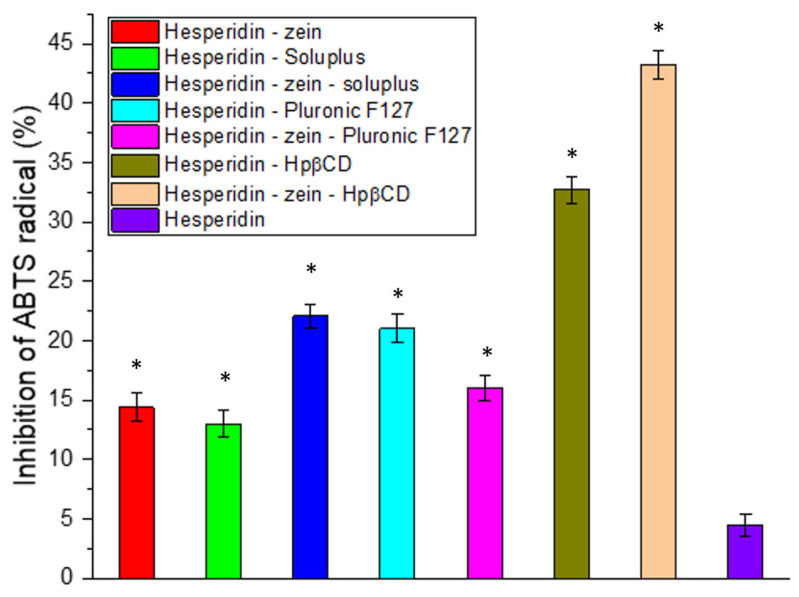
Antioxidant activity of hesperidin and obtained systems in the ABTS assay. Data are expressed as mean ± SD; *—significance with *p* ≤ 0.05.

Another critical activity expected from natural active substances is a high antioxidant activity [65,66]. For this reason, this activity was determined in the ABTS (Figure 3) and FRAP (Figure S4) models. In the case of the ABTS model used, we observed a significant improvement in the antioxidant capacity resulting from the increased concentration of HES. An almost 10-fold increase in the HES-zein-Hpβ-CD system activity was obtained compared to pure HES. However, despite the significant improvement in activity due to increased solubility, no IC_50_ values were obtained in the test system. In order to obtain the complete dependence of HES concentration and activity in the HES model, a test was performed to determine the IC_50_ value (Figure S2).

Moreover, in all tested systems, improvements in antioxidant properties were found, resulting from an increase in the concentrations of HES, thereby confirming the positive biological response as resulting from increasing the solubility of HES.

The FRAP model was used, which also determined the antioxidant potential of the obtained systems and the relationship between the concentration of HES and activity. The results indicated a linear relationship between the concentration and the obtained antioxidant activity (R^2^ = 0.9822), indicating that the increase in activity was intertwined with the increase in HES concentration (Figure S3).

Moreover, in the tested FRAP model, inhibition of HES was demonstrated, allowing the determination of the IC_0.5_ parameter at the level of 0.175 mg/mL. However, the demonstrated linear correlation between concentration and activity indicated that the concentration of HES determined the activity [67].

Thus, the obtained results allowed us to select the system with the highest biological activity potential resulting from the best improvement in HES solubility. In further research, the triple system consisting of HES-zein-Hpβ-CD was selected as having the best properties; however, to obtain complete characteristics, double HES systems with zein and Hpβ-CD were also taken into account as reference points, to create a complete picture of the changes taking place.

**Figure 4:**
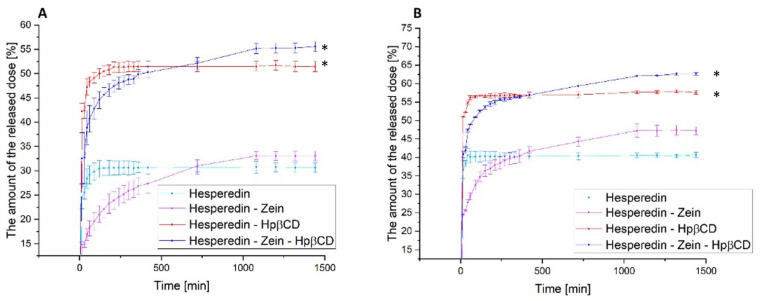
The dissolution rate of hesperidin and systems: hes-zein, hes-Hpβ-CD, hes-zein-Hpβ-CD in pH 1.2 (**A**) and 6.8 (**B**). Data are expressed as mean ± SD; *—significance with *p* ≤ 0.05.

**Figure 5:**
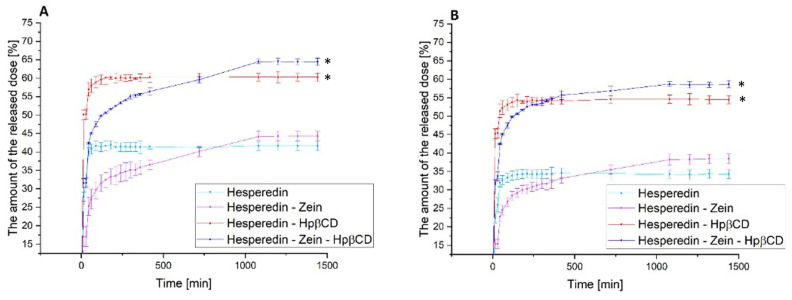
The dissolution rate of hesperidin and systems: hes-zein, hes-Hpβ-CD, hes-zein-Hpβ-CD in fasted state simulated intestinal fluid (**A**) and fed state simulated intestinal fluid (**B**). Data are expressed as mean ± SD; *—significance with *p* ≤ 0.05.

The next stage of the research was the analysis of the dissolution rate in classical media simulating subsequent sections of the gastrointestinal tract at pH 1.2 and 6.8 (Figure 4) and the determination of the relationship between the dissolution rate and the moment of administration; for this purpose, a model simulating the fasted state simulated intestinal fluid and fed state simulated intestinal fluid was used (Figure 5).

In the case of the pure HES release profile, we observed a virtually immediate achievement of the maximum compound dissolution level of about 30% at pH 1.2 and 40% at pH 6.8. The HES-Hpβ-CD binary system was characterized by equally high dynamics of compound dissolution with a simultaneous improvement in its solubility to 50% and 57% for gastric and intestinal pH, respectively. In the case of the zein-containing binary system, we observed an extended-release profile, reaching the plateau phase after about 18 h for both study conditions. This system was characterized by a slight improvement in the solubility of HES alone, up to 5% for pure HES. In the case of the triple HES system based on Hpβ-CD and zein, we observed the maintenance of two desirable solubilizer properties. The observed improvement in the solubility of HES, thanks to the use of Hpβ-CD with a solubility, was 52% and 65% for the stomach and intestine, with an extended-release profile with a plateau phase in about 18 h. Moreover, there was an extended-release profile, with a plateau phase after about 18 h of the process. A system with improved solubility was obtained, while obtaining a sustained release profile of HES.

The next stage of the research was to determine the effect of food consumption on the bioavailability of HES. This analysis was carried out due to the well-known effect of food consumption on the bioavailability of polyphenols [15,68,69]. Thus, a model simulating the gut in a state of hunger and the gut after food consumption was used. The obtained results confirmed the maintenance of the previously observed trends for the tested systems. Moreover, the obtained results indicated a 5–10% improvement in the release of the therapeutic dose of HES in the case of the hungry intestine as compared to the fed intestine. Of course, these were results obtained in the model only simulating the conditions in the gastrointestinal tract; however, they represented a valuable observation, indicating a significant impact of food consumption on the availability of the tested HES. Thus, it may be beneficial to take HES on an empty stomach, due to the improvement in its solubility, which should translate into an increase in the biological effect.

**Figure 6:**
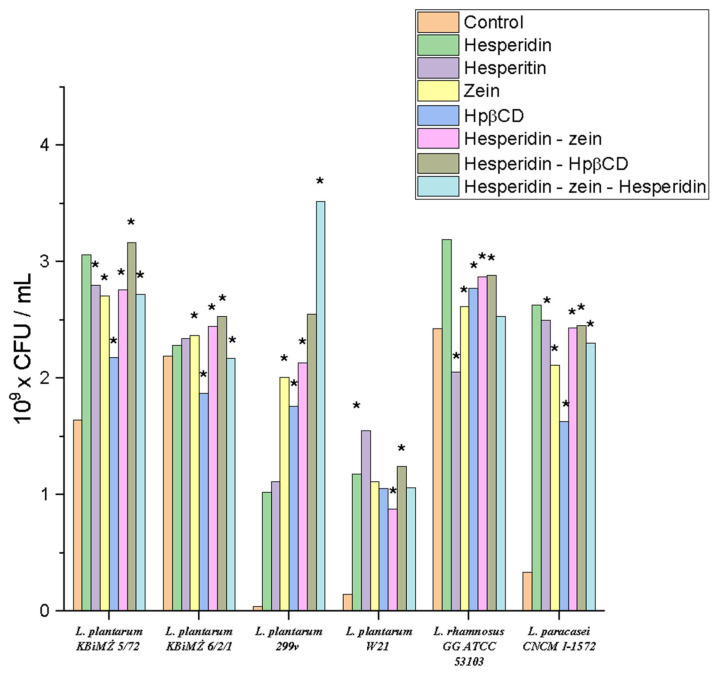
Screening tests of the influence of the obtained system on the growth of selected probiotic strains, *—significance with *p* ≤ 0.05.

The subsequent research stage determined the relationship between HES and selected probiotic strains representing the natural intestinal microbiota. For this purpose, the best obtained systems and their components were added to the liquid cultures containing the selected strain, to determine their prebiotic potential. In the conducted study, the activity of the final system was checked with all components separately, to evaluate the final effect. In addition, culture samples were taken at the indicated time intervals and subjected to HPLC analysis, to determine the quantification of HES changes and potential transformation into HEP. The study was divided into two stages: in the first stage, a screening analysis was performed on six selected probiotic strains belonging to the *Lactiplantibacillus* and *Lacticaseibacillus* genera. Cultures were carried out in small volumes (2.0 mL). Samples were collected at one time point after 24 h incubation with the selected substance. The control sample consisted of bacteria only grown on the medium, without adding substances in the system (Figure 6). Moreover, in all trials, tests of HEP activity were additionally conducted in the case of HES metabolism.

The results showed that all tested strains obtained a positive prebiotic effect for the applied triple system. The applied culture media observed an increase in the cell count of these bacteria relative to the control sample. For the strain *Lactiplantibacillus plantarum* KBiMŻ 6/2/1, Hpβ-CD reduced the bacterial count from 2.18 × 10^9^ to 1.87 × 10^9^ CFU/mL. A similar situation was observed for the *Lacticaseibacillus rhamnosus* GG ATCC 53103 strain, where HEP slightly inhibited its growth from 2.42 × 10^9^ to 2.05 × 10^9^ CFU/mL. It was noticed that the influence of the studied systems was strain-dependent. The highest positive effect on growth dynamics was observed for the strain *Lactiplantibacillus plantarum* 299v, where there was an increase in the control at the level of 4.43 × 10^7^ to 3.52 × 10^9^ CFU/mL. In the case of the strain *Lactiplantibacillus plantarum* KBiMŻ 6/2/1, the lowest dynamics of changes under the influence of incubation with the tested substances were noticed. A synergistic effect of Hpβ-CD on the effect of HES on the growth of most tested strains was also found.

In all systems, we observed a positive response, in the form of increased growth under the influence of HES and zein. The increased growth under the influence of zein may have been related to its utilization as an easily digestible nitrogen source by the tested *Lactobacillaless* order. Thus, confirming the positive prebiotic effect on probiotic bacteria. Differences in effects within the *Lactiplantibacillus* genus may have been due to the individual response of the strains [70,71]. The tested strains were characterized by a positive response to HES, indicating their natural ability to metabolize this polyphenol; however, more extensive screening studies are required to confirm this theory.

Moreover, HPLC analysis was performed to verify the ability of the tested strains to convert HES into HEP. The analysis results are presented in Table 2 as the percentage of HES loss from the beginning of the incubation process. Interestingly, despite the observed decrease in HES concentration, the appearance of HEP was not observed in any of the samples. This means that despite the occurring metabolism of the active substance under study by the selected probiotic strains, they could not hydrolyze the sugar bond to utilize the sugar residue and use it as a source of energy. The ability of the *L. plantarum* 299v strain to metabolize HES was the greatest.

The presence of zein-Hpβ-CD increased the HES utilization rate. The loss of HES content could be attributed to a different metabolism, which requires further studies specifically targeted to this end. However, the presented results clearly show the interaction between the probiotic strains from the *Lactocaseibacillus* genus and HES. The observed prebiotic effect was probably due to the HES metabolism and the use of its degradation products as a nutritional source or growth stimulant. The presented differences may be related to differences in the optimal conditions for the growth of individual strains, which in turn may affect their metabolism. Moreover, the differences may have been due to variable metabolic pathways within the *Lactiplantibacillus* genus [72,73,74].

Interestingly, in the samples containing HEP, no changes in concentration were observed in the input content. This proved a more beneficial glycoside effect on the health-promoting gut lactic acid bacteria than aglycone. Thus, it is possible to conclude that a sugar residue was involved or used in their metabolism.

**Table 2:** Quantitative change in hesperidin concentration after the 24th hour of culture of the selected probiotic strains (%). Data are expressed as mean ± SD; *—significance with *p* ≤ 0.05.

Strain	HES	HES–Zein	HES-Hpβ-CD	HES–Zein–Hpβ-CD
*L. plantarum* KBiMŻ 5/72	−11.34 ± 0.11	−12.48 ± 0.09	−11.67 ± 0.10	−12.03 ± 0.05
*L. plantarum* KBiMŻ 6/2/1	−7.21 ± 0.09	−7.05 ± 0.02	−6.45 ± 0.07 *	−7.41 ± 0.05 *
*L. plantarum* 299v	−12.31 ± 0.04	−15.52 ± 0.07 *	−16.73 ± 0.04 *	−22.34 ± 0.04 *
*L. plantarum* W21	−8.99 ± 0.08	−9.15 ± 0.06	−9.00 ± 0.08	−9.79 ± 0.07 *
*L. rhamnosus* GG ATCC 53103	−6.33 ± 0.04	−5.14 ± 0.02 *	−5.78 ± 0.06 *	−6.46 ± 0.06
*L. paracasei* CNCM I-1572	−10.98	−13.22 *	−13.01 *	−12.89 *

**Figure 7:**
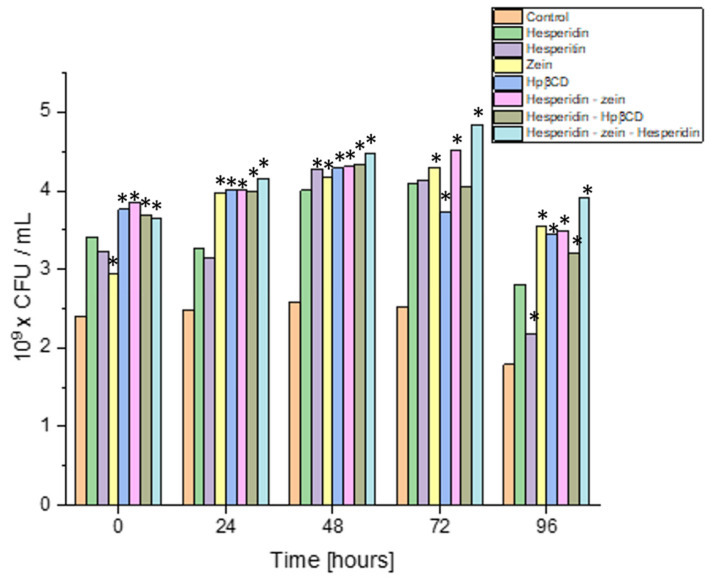
Growth of the *Lactiplantibacillus plantarum* 299v strain exposed to HES and the components of the tested triple system, *—significance with *p* ≤ 0.05.

The next step was to scale the process for the strain characterized by the highest dynamics of growth changes under the influence of the tested system. In order to increase the scale, the experiment was performed in 200 mL flask cultures, keeping the original proportions between the amount of the inoculum and the test system. Moreover, the performed analysis was carried out at four culture points, to accurately estimate the dynamics of the quantitative changes (Figure 7).

The results indicated that all substances used improved the growing intensity of the *Lactiplantibacillus plantarum* 299v strain. In control cultures, this strain grew up to 24 h, and then the number of its cells decreased slightly. All the additives lengthened the logarithmic growth phase of *L. plantarum* 299v by at least 24 h. Interestingly, this strain grew up to 72 h of culture in the three-component system. This system also yielded the highest biomass (4.85 × 10^9^ CFU/mL vs. 2.51 × 10^9^ CFU/mL in control). This may have resulted from the *L. plantarum* 299v strain using the sugar residue of the HES and nitrogen from the zein, which allowed the culture to be kept in a better condition for a longer time. The most remarkable change in cell count (density) was observed on the third day of culture.

Interestingly, all the substances tested showed a comparable prebiotic effect at that time, with a slight predominance of the triple system. This confirmed the originally obtained screening test results (or similar observations were made during screening tests). The experiment showed the most significant prebiotic effect with the obtained triple system. An essential aspect of the conducted study was that the positive influence of, not only the components of the tested system itself, but also the tested triple system, was proven.

**Figure 8:**
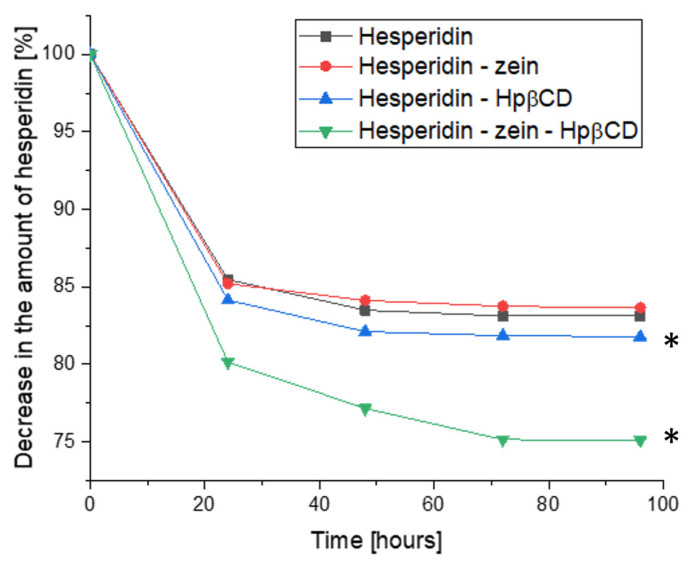
Quantitative change in hesperidin during the cultivation period as a loss of initial concentration (%), *—significance with *p* ≤ 0.05.

The change in HES concentration was also evaluated; this time, the analysis considered the dynamics of the concentration change during the cultivation (Figure 8). The results showed a decrease in HES concentration at consecutive sampling points. In this study, the formation of HEP as a metabolite was also not observed. This ruled out the influence of the time of cultivation on the conversion process. Moreover, it was confirmed that the HEP concentration did not change in the samples containing it; therefore, these results are not presented in a graphical form.

The HES concentration decreased during the logarithmic growth phase of *L. plantarum* 299v. This allowed the conclusion that the HES metabolism/utilization process is closely related to the growth of bacterial cells, which thus confirmed the assumption about the possibility of using HES as a growth factor or nutritional source for probiotic strains and other gut microbiota. The highest dynamics of change in HES concentration was observed during the first 24 h of cultivation. The HES concentration decreased up to 72 h, maintaining a plateau between 72 and 96 h, i.e., during the *L. plantarum* 266v stationary growth phase. However, in the case of the triple system, the dynamics of the decrease in HES concentration were significantly greater for 48 and 72 h compared to binary systems and pure HES. This proved the improved availability of HES for bacterial culture, by providing it as a developed triple system.

The interaction between antioxidant compounds, such as hesperidin, and probiotic survival in the gut microbiome can have positive implications. Antioxidants, known for their ability to scavenge free radicals and reduce oxidative stress, play a crucial role in protecting probiotics and enhancing their viability [75]. Oxidative stress can be detrimental to probiotic cells, hindering their survival and functionality. However, the presence of antioxidant compounds, such as hesperidin, creates a favorable environment, by shielding probiotics from oxidative damage. This protection ensures their colonization and activity within the gut, contributing to their overall effectiveness [76].

Moreover, antioxidant compounds have been shown to promote a balanced gut microbiome. By reducing oxidative stress, antioxidants help maintain the diversity and stability of the gut microbial community [77]. A balanced microbiome is essential for optimal gut function and overall well-being. Thus, the presence of antioxidant compounds indirectly contributes to the preservation of probiotic populations within the gut microbiome. Antioxidants and probiotics have independent health benefits, but their combined effects can exhibit synergistic advantages for gut health [78]. Antioxidant compounds, including hesperidin, possess anti-inflammatory properties, mitigating inflammation within the gut. This anti-inflammatory environment provides favorable conditions for probiotics to thrive and exert their beneficial effects. Additionally, certain antioxidants may enhance the adhesion and colonization of probiotics in the gut, further improving their survival and efficacy [79]. To maximize the benefits of antioxidants on probiotic viability, finding the optimal concentrations and formulations is crucial. Extremely high concentrations of antioxidants may pose challenges to probiotic survival, highlighting the importance of balance.

## 3. Materials and Methods

### 3.1. Materials and Instruments

Standard compounds used in the HPLC analysis: Hesperidin (>95%) and Hesperetin (>95%) were supplied by Sigma-Aldrich, St. Louis, MO, USA.

Substances used in the preparation of systems: hesperidin (>95%), zein, 2-hydroxypropyl-β-cyclodextrin (molar substitution 0.8), Pluronic^®^ F-127 (Poloxamer 407) were obtained from Sigma Aldrich Chemie (Berlin, Germany), with Soluplus^®^ (PVCL-PVA-PEG) from BASF ChemTrade GmbH (Ludwigshafen, Germany).

Reagents used in the biological activity studies, including α-D-glucopyranoside (PNPG), α-glucosidase from Saccharomyces cerevisiae (Type I, lyophilized powder, ≥10 units/mg protein), acarbose, 2,2-diphenyl-1-picrylhydrazyl, TPTZ (2,4,6-tripyridyl-S-triazine), and iron (III) chloride hexahydrate (FeCl_3_·6H_2_O) were supplied by Sigma-Aldrich, St. Louis, MO, USA.

Other chemical reagents, including hydrochloric acid, dimethyl sulfoxide, sodium chloride, potassium dihydrogen phosphate, and sodium hydroxide, were obtained from Avantor Performance Materials (Gliwice, Poland), while FaSSIF/FeSSIF/FaSSGF powder simulating the effect of food was supplied by Biorelevant (London, UK).

For the microbiological studies, the following strains were assessed:

1. *Lactiplantibacillus plantarum* KBiMŻ 5/72-oscypek isolate from our own collection, GenBank deposit no. JF733814
2. *Lactiplantibacillus plantarum* KBiMŻ 6/2/1-gołka isolate from our own collection, GenBank deposit no. JF733805
3. *Lactiplantibacillus plantarum* 299v-isolate from the commercial product SanProbi IBS
4. *Lactiplantibacillus plantarum* W21-isolate from the commercial product SanProbi SuperFormuła
5. *Lacticaseibacillus rhamnosus* GG ATCC 53103
6. *Lacticaseibacillus paracasei* CNCM I-1572

All strains were stored in a Cryobank (Bacteria storage system, MAST Diagnostica, Reinfeld, Germany) at −20 °C. Before the studies, the strains were defrosted and passaged twice for regeneration in MRS broth (OXOID), the standard medium for *Lactobacillus* bacteria. Cultures were incubated/carried out at 37 °C for 24 h under anaerobic conditions.

The organic solvent evaporation process was carried out using incubator MaxQ 4450 (Thermo Scientific, Waltham, MA, USA). Qualitative and quantitative research was conducted using the high-performance liquid chromatograph Prominance-I LC-2030C and a UV detector, a plate reader (Multiskan GO (Thermo Scientific), and a laboratory incubator (MaxQ 4450, Thermo Scientific). To measure weight, a Radwag AS 220.X2 (Radom, Poland) analytical balance was used throughout the study.

### 3.2. Development of the Hesperidin-Solubilizer Systems

Preparation of the systems used for the tests was carried out using solvent evaporation. As the base solubilizer, zein was chosen due to its proven ability to extend the release rate and the solubility of sparingly soluble active substances. Moreover, three solubilizers with proven solubility improvement properties for sparingly soluble active substances were selected (Soluplus, Pluronic F-127, and (2-Hydroxypropyl)-β-cyclodextrin).

Then, 100.0 mL of 70% methanol (aqueous solution) was added to a conical flask with a flat bottom; afterward, 100.0 mg of zein and 100.0 mg of the second tested solubilizers (Soluplus, Pluronic F-127, or (2-Hydroxypropyl)-β-cyclodextrin) were added or, in the case of binary systems, one of the tested solubilizers in the amount of 100.0 mg (Soluplus, Pluronic F-127 or (2-Hydroxypropyl)-β-cyclodextrin). A 1:1:1 mass ratio between HES, zein, and solubilizer was used to prepare the systems. The applied mass ratio in the tested systems was maintained at one selected level, due to the effect of solubilizers belonging to different groups of chemical compounds. The use of the optimal mass ratio between successive substances was not determined in the studies. The prepared flask was placed on a magnetic stirrer, and the mixing process was carried out until the solubilizers had dissolved at a temperature of 25 °C and a stirring speed of 500 rpm. At the same time, a second 250 mL flat bottom conical flask was prepared to contain 100 mL of methanol, in which 100.0 mg of hesperidin was dissolved. After the contents of both flasks had dissolved, the flask containing the hesperidin solution was slowly poured into the flask containing the solubilizer solution. The prepared mixture of hesperidin and solubilizers solution was placed on a magnetic stirrer and stirred for 60 min at a temperature of 25 °C and stirring speed of 500 rpm. After the mixing process, the solution was transferred to a crystallizer and subsequently placed in an incubator for 48 h at 40 °C with continuous stirring at 100 rpm. After the solvent evaporation process, the crystallizer’s content was homogenized with an electric grinder. As a result of the preparation, 7 systems were obtained:

1. Hesperidin-zein
2. Hesperidin-Soluplus
3. Hesperidin-zein-Soluplus
4. Hesperidin-Pluronic F-127
5. Hesperidin-zein-Pluronic F-127
6. Hesperidin-2-Hydroxypropyl-β-cyclodextrin
7. Hesperidin-zein-2-Hydroxypropyl-β-cyclodextrin

As a result, binary systems containing each tested solubilizer, triple systems containing zein as the base carrier, and one previously selected solubilizer were obtained.

### 3.3. Development of the HPLC Method

In order to carry out a quantitative analysis, as part of the experiments performed, an HPLC method was developed that allowed for the simultaneous separation of hesperidin and its hesperetin aglycone (HEP), which could have appeared as contamination in the obtained samples.

Chromatographic analysis was performed using a Shimadzu Prominance LC-2030C apparatus with a UV detector (Shimadzu Corp., Kyoto, Japan). The isocratic method developed was based on using an Eclipse Plus C18 column (4.6 × 250; 5 µm) (Agilent Technologies, Santa Clara, CA, USA) with the oven thermostat set to 35 °C. The mobile phase was 0.1% trifluoroacetic acid and methanol in a 1:1 ratio at a 1 mL/min flow, the detection was carried out at 270 nm, and 10 µL of the test solution was injected. The analysis was carried out for 18 min. In terms of selectivity, linearity, intra- and inter-day accuracy, limits of detection (LOD), and quantitation, the HPLC-DAD method was validated according to the International Conference on Harmonization Guideline Q2 (LOQ) [80].

In order to test the HES content in the obtained systems, 30 mg of the system was weighed into a falcon tube, and 30 mL of methanol was added. The sample prepared in this way was placed for 30 min in an ultrasonic bath at 30 °C. After extraction of the HES from the system, the sample was filtered through a 0.22 µm nylon syringe filter.

After the dissolution and dissolution rate tests, samples were filtered through a nylon syringe filter with a pore diameter of 0.22 µm. The samples, after microbiological culture, were centrifugated before filtration, to sediment the biomass in the sample.

### 3.4. Powder X-ray Diffraction (PXRD)

PXRD analysis was performed at ambient temperature using a Bruker D2 Phaser (Bruker, Billerica, MA, USA) diffractometer with a LynxEye XE-T 1-dim detector and Cu Kα radiation (λ = 1.54056 Å, generator setting: 40 kV and 40 mA). Diffraction data were collected at the 2θ scanning range between 5° and 40° with a step size of 0.02° and 00a counting time of 2 s/step.

### 3.5. Fourier Transform Infrared Spectroscopy (FT-IR)

The FT-IR–ATR spectra of hesperidin, zein, Soluplus, Pluronic F127, and Hpβ-CD, as well as their double and triple systems, were obtained using an IRTracer-100 spectrophotometer (Shimadzu Corp.). All spectra were measured between 400 and 4000 cm^−1^ in absorbance mode. The following spectrometer parameters were used: resolution: 4 cm^−1^, number of scans: 400, apodization: Happ–Genzel. The sample was placed directly on the ATR crystal. Solid samples were pressed against the ATR crystal, and the ATR–FT-IR spectrum was measured.

### 3.6. Solubility

In order to characterize the obtained systems, a dissolution test was performed. First, 50 mL of water was added to a 100 mL flat-bottom conical flask. Then, one of the obtained systems was added to each flask, and pure hesperidin was added to the last flask as a control. The flasks prepared in this way were tightly closed, to reduce water evaporation and placed in an incubator at 25 °C for 24 h with continuous stirring at 300 rpm. After incubation, 2.0 mL of the solution was withdrawn from each flask and filtered into the vial using a nylon syringe filter with a pore diameter of 0.22 µm. The obtained samples were analyzed by HPLC using the previously described method.

All determinations were performed in 3 independent tests; 3 independent samples were taken from each, so each system was subjected to 9 HPLC analyses.

### 3.7. In Vitro Activity of Systems

#### 3.7.1. α-Glucosidase Inhibition Assay

A spectrophotometric method with minor modifications was used to determine the inhibition of α-glucosidase by the hesperidin solutions based on the concentration obtained in the solubility test for each of the obtained systems and on the pure substance [81]. Briefly, 50.0 μL of sample solution (hesperidin concentration obtained in the solubility test prepared in a 30% DMSO solution for subsequent systems and pure test substance) or acarbose (positive control, 1–5 mg/mL), 50.0 μL of 0.1 M phosphate buffer (pH 6.8), and 30.0 µL α-glucosidase solution (1.0 U/mL) was pre-incubated in 96-well plates at 37 °C for 15 min. Next, 20.0 μL of 5 mM p-nitrophenyl-α-D-glucopyranoside (pNPG) solution in a 0.1 M phosphate buffer (pH 6.8) was added and incubated at 37 °C for 20 min. The reaction was terminated by adding 100.0 µL of sodium carbonate (0.2 M) to the mixture. The absorbance of the liberated p-nitrophenol was measured at 405 nm. The absorbance of enzyme solution, but without hesperidin solution/acarbose, served as a control with total enzyme activity. The absorbance in the absence of the enzyme was used as a blind control. The enzyme inhibition rate, expressed as a percentage of inhibition, was calculated using the following formula:

% inhibition activity = ((A_C_ − A_S_)/A_C_) × 100

where

A_C_ is the absorbance of the control (100% enzyme activity),

A_S_ is the absorbance of the tested sample.

For the investigated solutions, two independent experiments were carried out in triplicate. Results were expressed as means ± S.D.

#### 3.7.2. ABTS (2,2′-Azino-bis(3-ethylbenzothiazoline-6-sulfonic acid) Assay

A spectrophotometric method was used to determine the antioxidant activity, performed according to Re et al., with modifications [82]. The green cation radical was generated upon the loss of an electron by the nitrogen atom of ABTS caused by potassium persulfate. After introducing a pre-formed radical cation to the antioxidant, the ABTS radical cation was reduced and converted to its colorless neutral form. The HES concentrations for assay were prepared according to the obtained dissolutions in DMSO. The assay was performed on a 96-well plate with 50.0 µL of the previously prepared HES solutions and 200.0 µL of the ABTS^•+^ solution applied to the wells. The plate was incubated with shaking for 10 min at room temperature. After the incubation, the absorbance values were measured at λ = 734 nm. Vitamin C was used as a standard. The percentage inhibition of the ABTS^•+^ by the samples was calculated according to the following equation:

ABTS scavenging activity (%) = *A*_0_ − *A*_1_*A*_0_ × 100%

where

*A*_0_ is the absorbance of the control;

*A*_1_ is the absorbance of the sample.

#### 3.7.3. Ferric Reducing Antioxidant Power Assay (FRAP)

Following Tiveron et al., a FRAP assay was performed with some modifications [83]. The stock solutions of FRAP reagent included 300 mM acetate buffer (pH 3.6), 10 mM TPTZ solution in 40 mM HCl, and 20 mM FeCl_3_·6H_2_O solution. The working FRAP solution was freshly prepared by mixing 25 mL of acetate buffer, 2.5 mL of TPTZ solution, and 2.5 mL of FeCl_3_·6H_2_O solution and then warmed at 37 °C before usage. Briefly, 25.0 μL of the tested solutions of hesperidin concentration obtained in the solubility test and pure substance were mixed with 175.0 μL of FRAP solution, shaken, and incubated at 37 °C for 30 min in the dark. Then the absorbance was read at 593 nm. The results were presented as the obtained absorbance of the sample with a given concentration at the wavelength of 593 nm, where a higher value indicated a higher activity of the tested concentration.

### 3.8. Dissolution Rate Studies

A dissolution rate analysis was performed for the triple system with the best solubility and thus the highest in vitro activity in the tests (System 7. HES-zein-Hpβ-CD). Pure HES was used as reference samples and double systems number 1 (hesperidin-zein) and number 6 (hesperidin-Hpβ-CD), components of system 7.

The change in dissolution rate of the obtained hesperidin systems was examined by determining the dissolution rate profiles, following the requirements of the European Pharmacopoeia at 37 ± 0.5 °C, using a paddle apparatus (Agilent, Santa Clara, CA, USA) with a paddle rotation speed of 50 rpm. As the acceptor dissolution media, 0.1 mol/L hydrochloric acid (pH~1.2) and phosphate buffer (pH~6.8) were used in the pH range corresponding to the gastrointestinal tract environment in 500.0 mL. In addition, two other non-standard media with pH 6.8 were used to simulate fasting and postprandial gut conditions using FaSSIF/FeSSIF/FaSSGF powder (Biorelevant, London, UK) in a volume of 500.0 mL. Hesperidin and the obtained systems were weighed into gelatin capsules and placed in a sinker, to prevent capsule flotation on the liquid’s surface. Dissolution samples were taken at appropriate time points. The samples were filtered through 0.22 μm nylon membrane syringe filters.

Dissolution profiles were compared using the model proposed by Moore and Flanner, which is based on two-factor values, difference *f*_1_ and similarity *f*_2_ [84], according to the formulas below:

$$f_{1}=\frac{\sum_{j=1}^{n}\left|R_{j}-T_{j}\right|}{\sum_{j=1}^{n}R_{j}}\times 100$$

$$f_{2}=50\times \log\left({\left(1+\left(\frac{1}{n}\right)\overset{n}{\underset{j=1}{\sum }}{\left|R_{j}-T_{j}\right|}^{2}\right)}^{-\frac{1}{2}}\times 100\right)$$

where

*n* is the sampling number,

*R_j_* and *T_j_* are the percent dissolved of the reference (HES) and test system at each time point.

Release profiles are assumed to be similar when *f*_1_ is between 0 and 15, while *f*_2_ is close to 100 (not less than 50).

### 3.9. The Interaction of the System with the Selected Gut Bacteria (or Probiotic Bacteria)

Cultures of regenerated probiotic strains were centrifuged at 4500× *g* for 10 min, and cells were washed in sterile physiological saline. Then, cell suspensions were adjusted to 0.5 on the McFarland scale (1.5 × 10^8^ CFU/mL) using a McFarland Densitometer (Biosan, Saratoga Springs, NY, USA) and used for inoculation 2 mL of standard MRS broth medium (being the control) and MRS medium, with addition single component system HES, HEP, zein and HpβCD, the binary system composed of HES–zein and HES-HpβCD, and the triple system composed of HES-zein-Hpβ-CD. The components above were added in the maximum amount soluble in the MRS medium, without any adverse effects on the tested bacteria strains established experimentally during preliminary studies. The initial concentration of bacteria in all media was adjusted to a level of 1.5 × 10^5^ CFU/mL. The incubation was conducted without pH regulation at 37 °C for 24 h. After incubation, the viable cells count was determined using Koch’s plate method. Collected samples were decimally diluted in sterile physiological saline, plated on Petri dishes with MRS agar (OXOID), and incubated at 37 °C for 48–72 h. The colonies used for evaluating viable cell numbers were counted using an automatic colony counter (Easy Count 2) and expressed in CFU/mL. The strain that responded the best to the tested/obtained systems was cultured on a 200.0 mL scale for 96 h in the same conditions. During incubation, samples were taken to evaluate the viable cells count at 0, 24, 48, 72, and 96 h. The results were given as colony-forming units (CFU/mL). The tests were run in triplicate.

### 3.10. Statistical Analysis

Statistical analysis was performed using Statistica 13.3 software (TIBCO Software Inc., Palo Alto, CA, USA). A Shapiro–Wilk test was implemented, to check the data distribution normality. Statistical significance was performed using a one-way analysis of variance (ANOVA), followed by Tukey’s HSD test. Measurements were considered significant between hesperidin and systems at *p* < 0.05.

## 4. Conclusions

As a result of the conducted research, it was proven that the zein in the tested systems acted as an effective carrier that provided an extended release profile of hesperidin from the delivery systems; in addition to hesperidin systems, solubilizers such as Soluplus, Pluronic F-127, and HpβCD increased hesperidin’s solubility. The highest solubility was obtained for the triple system containing cyclodextrin, and an over six-fold increase in solubility was observed. Notably, the improvement in the solubility of hesperidin correlated with a higher biological activity, considering the inhibition of α-glucosidase. Here, with a six-fold increase in solubility, an over 10-fold increase in activity was observed.

Moreover, an increase in antioxidant activity was shown; depending on the model, the pattern was exponential (ABTS) or showed a linear relationship (FRAP). Moreover, the obtained systems stimulated the growth of selected probiotic bacteria belonging to *Lactiplantibacillus* genus. Thus, zein as a functional carrier, together with solubilizers, turned out to significantly change the solubility of hesperidin; and at the same time, it was confirmed that it did not limit its biological activities, even inducing their growth.

## Figures and Tables

**Table 1:** Solubility of hesperidin and the obtained systems (after 24 h).

System	Solubility [mg/mL]
1. Hesperidin-zein	0.0797 ± 1 × 10^−4^
2. Hesperidin-Soluplus	0.0765 ± 1 × 10^−4^ *
3. Hesperidin-zein-Soluplus	0.1395 ± 2 × 10^−4^ *
4. Hesperidin-Pluronic F-127	0.1275 ± 3 × 10^−4^ *
5. Hesperidin-zein-Pluronic F-127	0.0771 ± 1 × 10^−4^ *
6. Hesperidin-Hpβ-CD	0.2053 ± 5 × 10^−4^ *
7. Hesperidin-zein-Hpβ-CD	0.3130 ± 5 × 10^−4^ *
8. Hesperidin	0.0499 ± 4 × 10^−4^ *

Data are expressed as mean ± SD; * significance with *p* ≤ 0.05.

## Data Availability

The data are contained within the article or Supplementary Materials.

## Supplementary Materials

The following supporting information can be downloaded at: https://www.mdpi.com/article/10.3390/molecules28135209/s1. Figure S1. Diffractograms of the obtained systems: A—zein; B—Soluplus; C—Pluronic F-127; D—Hpβ-CD; Figure S2. FT-IR spectrum for hesperidin, zein, Soluplus, Pluronic F127, Hpβ-CD, and the binary and triple systems were obtained; Figure S3. α-glucosidase inhibition profile with IC 50 parameter determined; Figure S4. Profile of antioxidant activity in the ABTS model with the determined IC 50 parameter; Figure S5. Antioxidant activity of hesperidin and obtained systems in FRAP assay; Table S1. Log cycle values (CFU/mL) for screening cultures of selected bacterial strains; Table S2. Log cycle values (CFU/mL) for *Lactiplantibacillus plantarum* 299v culture conducted for 96 h.

## Sample Availability

Not available.

## References

[1] Huang W., Long C., Lam E. (2018). Roles of Plant-Associated Microbiota in Traditional Herbal Medicine. Trends Plant Sci..

[2] Pferschy-Wenzig E.-M., Pausan M.R., Ardjomand-Woelkart K., Röck S., Ammar R.M., Kelber O., Moissl-Eichinger C., Bauer R. (2022). Medicinal Plants and Their Impact on the Gut Microbiome in Mental Health: A Systematic Review. Nutrients.

[3] Cheung M.K., Yue G.G.L., Chiu P.W.Y., Lau C.B.S. (2020). A Review of the Effects of Natural Compounds, Medicinal Plants, and Mushrooms on the Gut Microbiota in Colitis and Cancer. Front. Pharmacol..

[4] Wani S.U.D., Kakkar V., Gautam S.P., Hv G., Ali M., Masoodi M.H., Moin A. (2021). Enhancing Therapeutic Potential of Poor Aqueous Soluble Herbal Drugs through Solid Dispersion-An Overview. Phytomed. Plus.

[5] Zeng X., Yang X., Fan J., Tan Y., Ju L., Shen W., Wang Y., Wang X., Chen W., Ju D. (2021). MASI: Microbiota—Active Substance Interactions Database. Nucleic Acids Res..

[6] Pan C., Guo Q., Lu N. (2019). Role of Gut Microbiota in the Pharmacological Effects of Natural Products. Evid.-Based Complement. Altern. Med..

[7] An X., Bao Q., Di S., Zhao Y., Zhao S., Zhang H., Lian F., Tong X. (2019). The Interaction between the Gut Microbiota and Herbal Medicines. Biomed. Pharmacother..

[8] Spisni E., Turroni S., Shahaj S., Spigarelli R., Ayala D., Valerii M.C. (2020). Natural Compounds in the Modulation of the Intestinal Microbiota: Implications in Human Physiology and Pathology.

[9] Zheng Y., Gou X., Zhang L., Gao H., Wei Y., Yu X., Pang B., Tian J., Tong X., Li M. (2020). Interactions Between Gut Microbiota, Host, and Herbal Medicines: A Review of New Insights Into the Pathogenesis and Treatment of Type 2 Diabetes. Front. Cell. Infect. Microbiol..

[10] Yu Z.-K., Xie R.-L., You R., Liu Y.-P., Chen X.-Y., Chen M.-Y., Huang P.-Y. (2021). The Role of the Bacterial Microbiome in the Treatment of Cancer. BMC Cancer.

[11] Gupta A., Saha S., Khanna S. (2020). Therapies to Modulate Gut Microbiota: Past, Present and Future. World J. Gastroenterol..

[12] Fong W., Li Q., Yu J. (2020). Gut Microbiota Modulation: A Novel Strategy for Prevention and Treatment of Colorectal Cancer. Oncogene.

[13] Gebrayel P., Nicco C., Al Khodor S., Bilinski J., Caselli E., Comelli E.M., Egert M., Giaroni C., Karpinski T.M., Loniewski I. (2022). Microbiota Medicine: Towards Clinical Revolution. J. Transl. Med..

[14] Wdowiak K., Walkowiak J., Pietrzak R., Bazan-Woźniak A., Cielecka-Piontek J. (2022). Bioavailability of Hesperidin and Its Aglycone Hesperetin—Compounds Found in Citrus Fruits as a Parameter Conditioning the Pro-Health Potential (Neuroprotective and Antidiabetic Activity)—Mini-Review. Nutrients.

[15] Manach C., Morand C., Gil-Izquierdo A., Bouteloup-Demange C., Rémésy C. (2003). Bioavailability in Humans of the Flavanones Hesperidin and Narirutin after the Ingestion of Two Doses of Orange Juice. Eur. J. Clin. Nutr..

[16] Li Y., Li X., Li G., Du W., Zhang J., Li W., Xu J., Hu M., Zhu Z. (2008). In Vivo Pharmacokinetics of Hesperidin Are Affected by Treatment with Glucosidase-like BglA Protein Isolated from Yeasts. J. Agric. Food Chem..

[17] Savjani K.T., Gajjar A.K., Savjani J.K. (2012). Drug Solubility: Importance and Enhancement Techniques. ISRN Pharm..

[18] Sareen S., Mathew G., Joseph L. (2012). Improvement in Solubility of Poor Water-Soluble Drugs by Solid Dispersion. Int. J. Pharm. Investig..

[19] Chaudhary N., Tripathi D., Rai A.K. (2020). A Technical Approach of Solubility Enhancement of Poorly Soluble Drugs: Liquisolid Technique. Curr. Drug Deliv..

[20] Guo X., Li K., Guo A., Li E. (2020). Intestinal Absorption and Distribution of Naringin, Hesperidin, and Their Metabolites in Mice. J. Funct. Foods.

[21] Nielsen I.L., Chee W., Bredsdorff L., Offord E., Rasmussen S., Frederiksen H., Enslen M., Barron D., Horcajada M.-N., Williamson G. (2006). Bioavailability Is Improved by Enzymatic Modification of the Citrus Flavonoid Hesperidin in Humans: A Randomized, Double-Blind, Crossover Trial1. J. Nutr..

[22] Nectoux A.M., Abe C., Huang S.-W., Ohno N., Tabata J., Miyata Y., Tanaka K., Tanaka T., Yamamura H., Matsui T. (2019). Absorption and Metabolic Behavior of Hesperidin (Rutinosylated Hesperetin) after Single Oral Administration to Sprague-Dawley Rats. J. Agric. Food Chem..

[23] Actis-Goretta L., Dew T.P., Lévèques A., Pereira-Caro G., Rein M., Teml A., Schäfer C., Hofmann U., Schwab M., Eichelbaum M. (2015). Gastrointestinal Absorption and Metabolism of Hesperetin-7-O-Rutinoside and Hesperetin-7-O-Glucoside in Healthy Humans. Mol. Nutr. Food Res..

[24] Estruel-Amades S., Massot-Cladera M., Pérez-Cano F.J., Franch À., Castell M., Camps-Bossacoma M. (2019). Hesperidin Effects on Gut Microbiota and Gut-Associated Lymphoid Tissue in Healthy Rats. Nutrients.

[25] Wilmsen P.K., Spada D.S., Salvador M. (2005). Antioxidant Activity of the Flavonoid Hesperidin in Chemical and Biological Systems. J. Agric. Food Chem..

[26] Hajialyani M., Hosein Farzaei M., Echeverría J., Nabavi S.M., Uriarte E., Sobarzo-Sánchez E. (2019). Hesperidin as a Neuroprotective Agent: A Review of Animal and Clinical Evidence. Molecules.

[27] Stanisic D., Costa A.F., Favaro W.J., Tasic L., Seabra A.B., Duran N. (2018). Anticancer Activities of Hesperidin and Hesperetin In Vivo and Their Potentiality against Bladder Cancer. J. Nanomed. Nanotechnol..

[28] Ahmed O.M., Mahmoud A.M., Abdel-Moneim A., Ashour M.B. (2012). Antidiabetic effects of hesperidin and naringin in type 2 diabetic rats. Diabetol. Croat..

[29] Cunningham A.L., Stephens J.W., Harris D.A. (2021). Gut Microbiota Influence in Type 2 Diabetes Mellitus (T2DM). Gut Pathog..

[30] Zhang S., Cai Y., Meng C., Ding X., Huang J., Luo X., Cao Y., Gao F., Zou M. (2021). The Role of the Microbiome in Diabetes Mellitus. Diabetes Res. Clin. Pract..

[31] Mas-Capdevila A., Teichenne J., Domenech-Coca C., Caimari A., Del Bas J.M., Escoté X., Crescenti A. (2020). Effect of Hesperidin on Cardiovascular Disease Risk Factors: The Role of Intestinal Microbiota on Hesperidin Bioavailability. Nutrients.

[32] Stevens Y., Rymenant E.V., Grootaert C., Camp J.V., Possemiers S., Masclee A., Jonkers D. (2019). The Intestinal Fate of Citrus Flavanones and Their Effects on Gastrointestinal Health. Nutrients.

[33] Cesar T., Fidelix M., Sivieri K., Millenkovic D. (2020). Daily Consumption of Orange Juice Modulated Intestinal Microbiota and Improved Glucose and Lipids Metabolism in Women. Proc. Nutr. Soc..

[34] Lima A.C.D., Cecatti C., Fidélix M.P., Adorno M.A.T., Sakamoto I.K., Cesar T.B., Sivieri K. (2019). Effect of Daily Consumption of Orange Juice on the Levels of Blood Glucose, Lipids, and Gut Microbiota Metabolites: Controlled Clinical Trials. J. Med. Food.

[35] Duque A.L.R.F., Monteiro M., Adorno M.A.T., Sakamoto I.K., Sivieri K. (2016). An Exploratory Study on the Influence of Orange Juice on Gut Microbiota Using a Dynamic Colonic Model. Food Res. Int..

[36] Park M., Choi J., Lee H.-J. (2020). Flavonoid-Rich Orange Juice Intake and Altered Gut Microbiome in Young Adults with Depressive Symptom: A Randomized Controlled Study. Nutrients.

[37] Shukla R., Cheryan M. (2001). Zein: The Industrial Protein from Corn. Ind. Crops Prod..

[38] Labib G. (2018). Overview on Zein Protein: A Promising Pharmaceutical Excipient in Drug Delivery Systems and Tissue Engineering. Expert Opin. Drug Deliv..

[39] Paliwal R., Palakurthi S. (2014). Zein in Controlled Drug Delivery and Tissue Engineering. J. Control. Release.

[40] El-Rashidy A.A., Waly G., Gad A., Roether J.A., Hum J., Yang Y., Detsch R., Hashem A.A., Sami I., Goldmann W.H. (2018). Antibacterial Activity and Biocompatibility of Zein Scaffolds Containing Silver-Doped Bioactive Glass. Biomed. Mater..

[41] De Marco I. (2022). Zein Microparticles and Nanoparticles as Drug Delivery Systems. Polymers.

[42] Korth N., Parsons L., Van Haute M.J., Yang Q., Hurst P., Schnable J.C., Holding D.R., Benson A.K. (2022). The Unique Seed Protein Composition of Quality Protein Popcorn Promotes Growth of Beneficial Bacteria From the Human Gut Microbiome. Front. Microbiol..

[43] Shin W., Kim S., Chun K.S. (1987). Structure of (R,S)-Hesperetin Monohydrate. Acta Cryst. C.

[44] Groom C.R., Bruno I.J., Lightfoot M.P., Ward S.C. (2016). The Cambridge Structural Database. Acta Cryst. B Struct. Sci. Cryst. Eng. Mater..

[45] Brown M.E., Glass B.D., Worthington M.S. (2002). Binary Systems of Nifedipine And Various Cyclodextrins in The Solid State. Thermal, FTIR, XRD Studies. J. Therm. Anal. Calorim..

[46] Mukne A.P. (2004). Triamterene-β-Cyclodextrin Systems: Preparation, Characterization and In Vivo Evaluation. AAPS PharmSciTech.

[47] Bersier P.M., Bersier J., Klingert B. (1991). Electrochemistry of Cyclodextrins and Cyclodextrin Inclusion Complexes. Electroanalysis.

[48] Kfoury M., Landy D., Fourmentin S. (2018). Characterization of Cyclodextrin/Volatile Inclusion Complexes: A Review. Molecules.

[49] Sambasevam K.P., Mohamad S., Sarih N.M., Ismail N.A. (2013). Synthesis and Characterization of the Inclusion Complex of β-Cyclodextrin and Azomethine. Int. J. Mol. Sci..

[50] Truzzi F., Tibaldi C., Zhang Y., Dinelli G., D′Amen E. (2021). An Overview on Dietary Polyphenols and Their Biopharmaceutical Classification System (BCS). Int. J. Mol. Sci..

[51] Kaur H., Kaur G. (2014). A Critical Appraisal of Solubility Enhancement Techniques of Polyphenols. J. Pharm..

[52] Anwer M.K., Al-Shdefat R., Jamil S., Alam P., Abdel-Kader M., Shakeel F. (2014). Solubility of Bioactive Compound Hesperidin in Six Pure Solvents at (298.15 to 333.15) K. J. Chem. Eng. Data.

[53] Berardi A., Bisharat L., Cespi M., Basheti I.A., Bonacucina G., Pavoni L., AlKhatib H.S. (2017). Controlled Release Properties of Zein Powder Filled into Hard Gelatin Capsules. Powder Technol..

[54] Jiao Y., Zheng X., Chang Y., Li D., Sun X., Liu X. (2018). Zein-Derived Peptides as Nanocarriers to Increase the Water Solubility and Stability of Lutein. Food Funct..

[55] Khalid S.H., Bashir M., Asghar S., Mallhi T.H., Khan I.U. (2019). Effect of Cyclodextrin Derivatization on Solubility and Efficacy of Drugs.

[56] Saokham P., Muankaew C., Jansook P., Loftsson T. (2018). Solubility of Cyclodextrins and Drug/Cyclodextrin Complexes. Molecules.

[57] Fenyvesi F., Nguyen T.L.P., Haimhoffer Á., Rusznyák Á., Vasvári G., Bácskay I., Vecsernyés M., Ignat S.-R., Dinescu S., Costache M. (2020). Cyclodextrin Complexation Improves the Solubility and Caco-2 Permeability of Chrysin. Materials.

[58] Zhu T., Zhang B., Feng Y., Li Z., Tang X., Ban X., Kong H., Li C. (2022). Beneficial Effects of Three Dietary Cyclodextrins on Preventing Fat Accumulation and Remodeling Gut Microbiota in Mice Fed a High-Fat Diet. Foods.

[59] Singh P., Medronho B., Valente A.J.M., Miguel M.G., Lindman B. (2018). Exploring the Prebiotic Effect of Cyclodextrins on Probiotic Bacteria Entrapped in Carboxymetyl Cellulose-Chitosan Particles. Colloids Surf. B Biointerfaces.

[60] Toro-Uribe S., Godoy-Chivatá J., Villamizar-Jaimes A.R., Perea-Flores M.d.J., López-Giraldo L.J. (2020). Insight of Polyphenol Oxidase Enzyme Inhibition and Total Polyphenol Recovery from Cocoa Beans. Antioxidants.

[61] Griffiths D.W. (1986). The Inhibition of Digestive Enzymes by Polyphenolic Compounds. Adv. Exp. Med. Biol..

[62] Akiyama S., Katsumata S., Suzuki K., Ishimi Y., Wu J., Uehara M. (2010). Dietary Hesperidin Exerts Hypoglycemic and Hypolipidemic Effects in Streptozotocin-Induced Marginal Type 1 Diabetic Rats. J. Clin. Biochem. Nutr..

[63] Peng P., Jin J., Zou G., Sui Y., Han Y., Zhao D., Liu L. (2021). Hesperidin Prevents Hyperglycemia in Diabetic Rats by Activating the Insulin Receptor Pathway. Exp. Med..

[64] Rekha S.S., Pradeepkiran J.A., Bhaskar M. (2019). Bioflavonoid Hesperidin Possesses the Anti-Hyperglycemic and Hypolipidemic Property in STZ Induced Diabetic Myocardial Infarction (DMI) in Male Wister Rats. J. Nutr. Intermed. Metab..

[65] de Mello Andrade J.M., Fasolo D., Watson R.R., Preedy V.R., Zibadi S. (2014). Chapter 20—Polyphenol Antioxidants from Natural Sources and Contribution to Health Promotion. Polyphenols in Human Health and Disease.

[66] Pandey K.B., Rizvi S.I. (2009). Plant Polyphenols as Dietary Antioxidants in Human Health and Disease. Oxid. Med. Cell. Longev..

[67] Lahmer N., Belboukhari N., Cheriti K., Sekkoum K. (2015). Hesperidin and Hesperitin Preparation and Purification from *Citrus sinensis* Peels. Pharma Chem..

[68] Bohn T. (2014). Dietary Factors Affecting Polyphenol Bioavailability. Nutr. Rev..

[69] Naumovski N., Blades B.L., Roach P.D. (2015). Food Inhibits the Oral Bioavailability of the Major Green Tea Antioxidant Epigallocatechin Gallate in Humans. Antioxidants.

[70] Song M., Yun B., Moon J.-H., Park D.-J., Lim K., Oh S. (2015). Characterization of Selected Lactobacillus Strains for Use as Probiotics. Korean J. Food Sci. Anim. Resour..

[71] Su Y., Chen X., Liu M., Guo X. (2017). Effect of Three Lactobacilli with Strain-Specific Activities on the Growth Performance, Faecal Microbiota and Ileum Mucosa Proteomics of Piglets. J. Anim. Sci. Biotechnol..

[72] Liu J., Hu D., Chen Y., Huang H., Zhang H., Zhao J., Gu Z., Chen W. (2018). Strain-Specific Properties of Lactobacillus Plantarum for Prevention of Salmonella Infection. Food Funct..

[73] Smetanková J., Hladíková Z., Valach F., Zimanová M., Kohajdová Z., Greif G., Greifová M. (2012). Influence of Aerobic and Anaerobic Conditions on the Growth and Metabolism of Selected Strains of *Lactobacillus plantarum*. Acta Chim. Slovaca.

[74] Gänzle M., Follador R. (2012). Metabolism of Oligosaccharides and Starch in Lactobacilli: A Review. Front. Microbiol..

[75] Riaz Rajoka M.S., Thirumdas R., Mehwish H.M., Umair M., Khurshid M., Hayat H.F., Phimolsiripol Y., Pallarés N., Martí-Quijal F.J., Barba F.J. (2021). Role of Food Antioxidants in Modulating Gut Microbial Communities: Novel Understandings in Intestinal Oxidative Stress Damage and Their Impact on Host Health. Antioxidants.

[76] Naliyadhara N., Kumar A., Kumar Gangwar S., Nair Devanarayanan T., Hegde M., Alqahtani M.S., Abbas M., Sethi G., Kunnumakkara A. (2023). Interplay of Dietary Antioxidants and Gut Microbiome in Human Health: What Has Been Learnt Thus Far?. J. Funct. Foods.

[77] Wang X., Qi Y., Zheng H. (2022). Dietary Polyphenol, Gut Microbiota, and Health Benefits. Antioxidants.

[78] Santhiravel S., Bekhit A.E.-D.A., Mendis E., Jacobs J.L., Dunshea F.R., Rajapakse N., Ponnampalam E.N. (2022). The Impact of Plant Phytochemicals on the Gut Microbiota of Humans for a Balanced Life. Int. J. Mol. Sci..

[79] Plamada D., Vodnar D.C. (2021). Polyphenols—Gut Microbiota Interrelationship: A Transition to a New Generation of Prebiotics. Nutrients.

[80] ICH Official Web Site: ICH. https://www.ich.org/page/quality-guidelines.

[81] Telagari M., Hullatti K. (2015). In-Vitro α-Amylase and α-Glucosidase Inhibitory Activity of Adiantum Caudatum Linn. and Celosia Argentea Linn. Extracts and Fractions. Indian J Pharm..

[82] Re R., Pellegrini N., Proteggente A., Pannala A., Yang M., Rice-Evans C. (1999). Antioxidant Activity Applying an Improved ABTS Radical Cation Decolorization Assay. Free Radic. Biol. Med..

[83] Tiveron A.P., Melo P.S., Bergamaschi K.B., Vieira T.M.F.S., Regitano-d’Arce M.A.B., Alencar S.M. (2012). Antioxidant Activity of Brazilian Vegetables and Its Relation with Phenolic Composition. Int. J. Mol. Sci..

[84] Moore J.W., Flanner H.H. (1996). Mathematical Comparison of Dissolution Profiles. Pharm. Technol..

