# Supplementary material for: Zein as an Effective Carrier for Hesperidin Delivery Systems with Improved Prebiotic Potential
Source: Molecules. 2023 Jul 4;28(13):5209. doi: 10.3390/molecules28135209 (PMC10343518; doi:10.3390/molecules28135209)
Supplement: Supplementary file 1 [file molecules-28-05209-s001.zip › molecules-2479986-supplementary.pdf]

molecules

MDPI

Article

Zein as an Effective Carrier for Hesperidin Delivery Systems with Improved Prebiotic Potential

Szymon Sip [1], Anna Sip [2], Andrzej Miklaszewski [3], Marcin Żarowski [4] and Judyta Cielecka-Piontek [1,*]

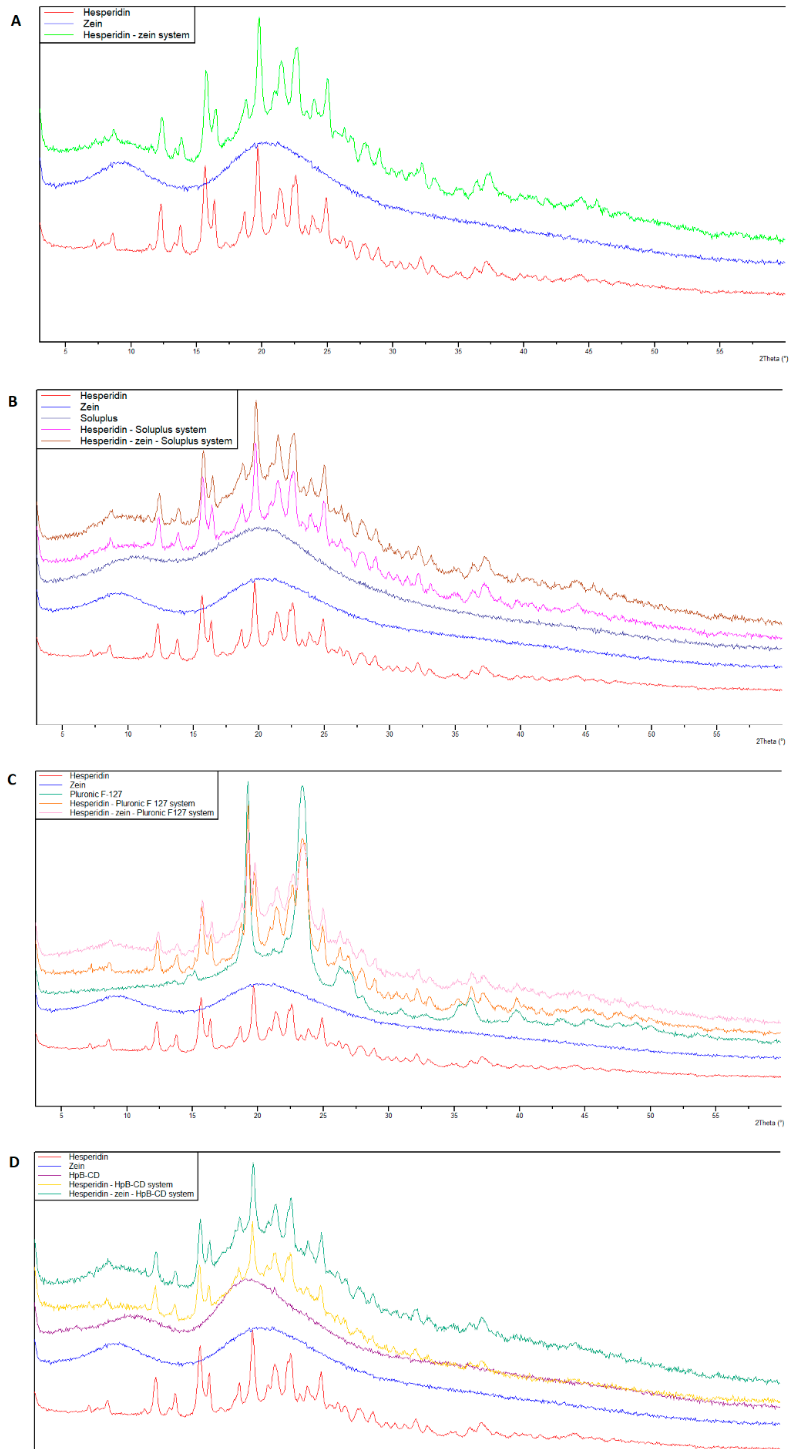

Figure S1. Diffractograms of the obtained systems: A - zein; B - Soluplus; C - Pluronic F-127; D - Hpβ-CD

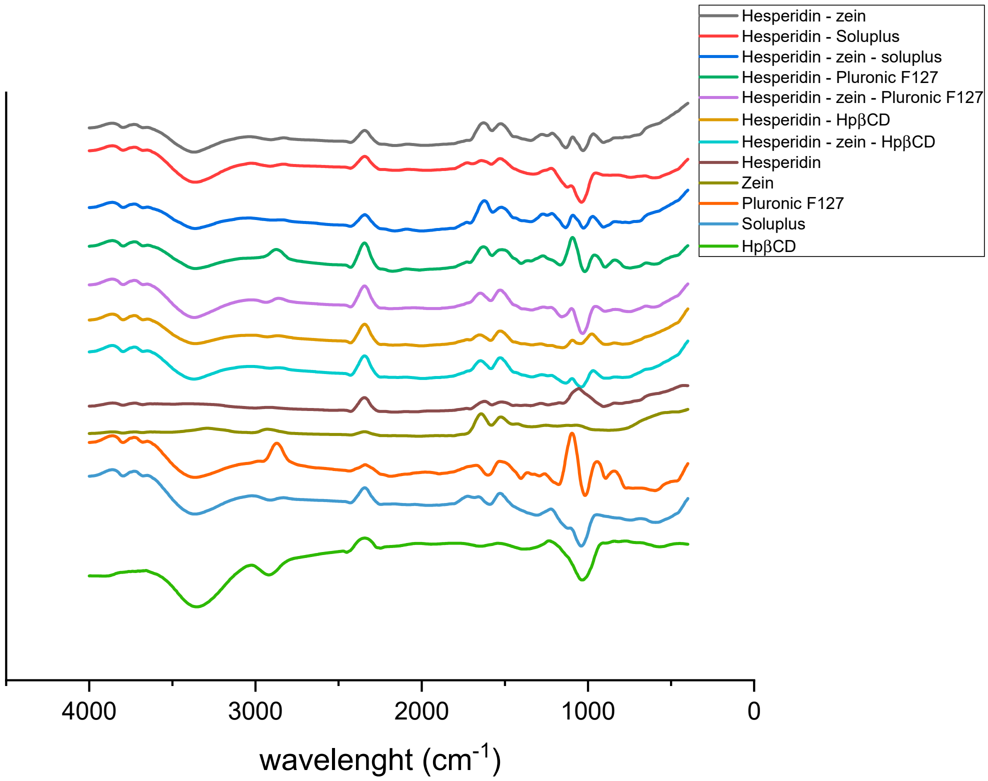

Figure S2. FT-IR spectrum for hesperidin, zein, Soluplus, Pluronic F127, Hpβ-CD, and the binary and triple systems were obtained**.**

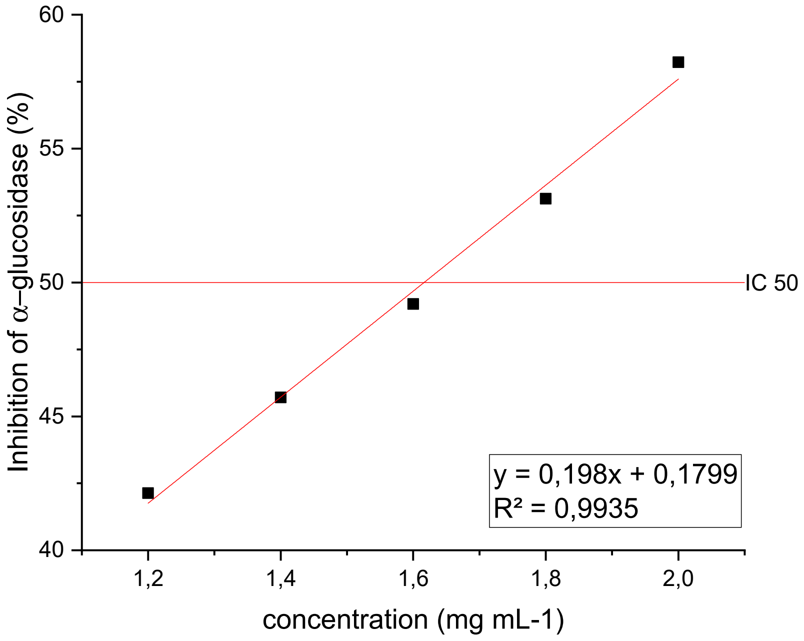

Figure S3. α -glucosidase inhibition profile with IC 50 parameter determined.

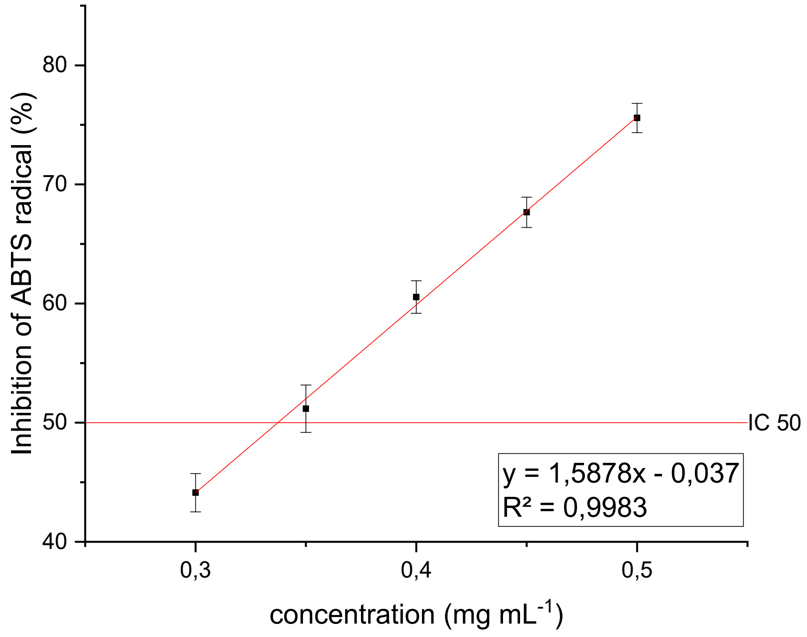

Figure S4. Profile of antioxidant activity in the ABTS model with the determined IC 50 parameter.

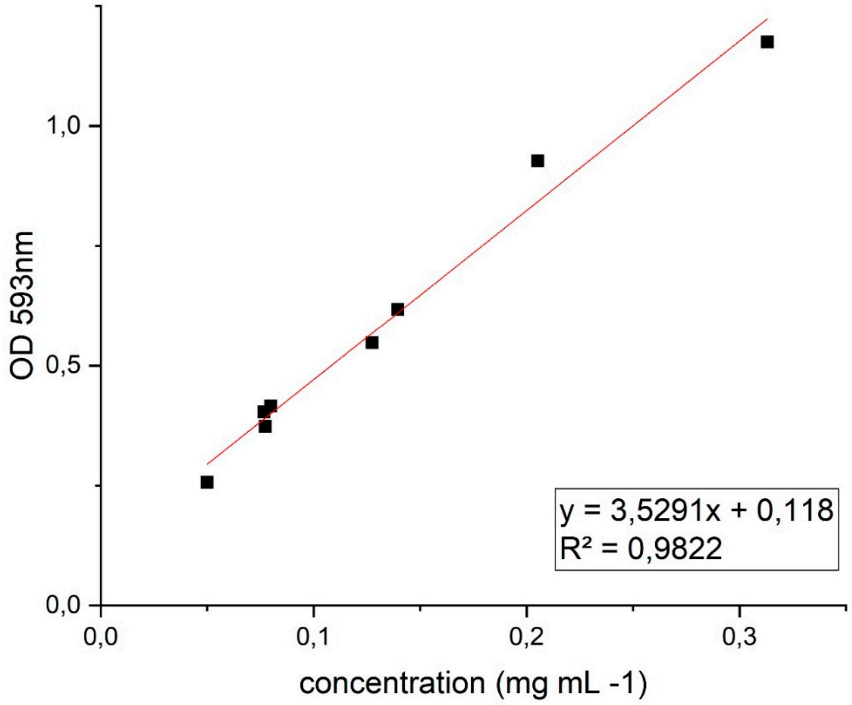

Figure S5. Antioxidant activity of hesperidin and obtained systems in FRAP assay.

Table S1. Log cycle values (CFU / ml) for screening cultures of selected bacterial strains

Bacterial strain	Control	Hesperidin	Hesperitin	Zein	HpβCD	Hesperidin - zein	Hesperidin - HpβCD	Hesperi-din - zein - HpβCD
L. plantarum KBiMŻ 5/72	9,216258	9,485588	9,447518	9,432682	9,338425	9,440717	9,50107	9,43499
L. plantarum KBiMŻ 6/2/1	9,34107	9,359338	9,369579	9,374648	9,272269	9,3884	9,403096	9,3371
L. plantarum 299v	7,646177	9,009332	9,046526	9,303226	9,246139	9,329097	9,406721	9,546334
L. plantarum W21	8,166667	9,072493	9,190158	9,046526	9,023615	8,943284	9,09459	9,025392
L. rhamnosus GG ATCC 53103	9,384676	9,504106	9,312852	9,417472	9,442994	9,458674	9,459777	9,403096
L. paracasei CNCM I-1572	8,528455	9,419837	9,398233	9,325066	9,2117	9,385921	9,389637	9,361911

Table S2. Log cycle values (CFU / ml) for *Lactiplantibacillus Plantarum* 299v culture conducted for 96h.

Time [H]	Control	Hesperidin	Hesperitin	Zein	HpβCD	Hesperidin - zein	Hesperidin - HpβCD	Hesperi-din - zein - HpβCD
0	9,447518	9,532422	9,509122	9,467438	9,576186	9,585065	9,566693	9,561495
24	9,395788	9,515067	9,496991	9,599293	9,603877	9,602358	9,602144	9,618574
48	9,412716	9,602358	9,630656	9,621407	9,632381	9,634089	9,63635	9,651422
72	9,465259	9,611337	9,617145	9,632381	9,57181	9,65381	9,606887	9,68533
96	9,253469	9,447518	9,3371	9,549056	9,536975	9,543591	9,507122	9,593112
